# OCT angiography analysis of retinal vessel density in primary open-angle glaucoma with and without Tafluprost therapy

**DOI:** 10.1186/s12886-020-01707-3

**Published:** 2020-11-12

**Authors:** Hannah Weindler, Martin S. Spitzer, Maximilian Schultheiß, Robert Kromer

**Affiliations:** Department of Ophthalmology, University Medical Center Hamburg-Eppendorf, Martinistraße 52, 20246 Hamburg, Germany

**Keywords:** Primary open-angle glaucoma, Optical coherence tomography angiography, Tafluprost, Retinal flow density, Optic nerve head, Macular region

## Abstract

**Background:**

Primary open-angle glaucoma (POAG) is a progressive neurodegenerative disease which leads to irreversible blindness. An elevated intraocular pressure (IOP) is considered to be the main risk factor for the disease progression. It is known that retinal blood flow is altered in POAG eyes. Tafluprost, a prostaglandin analogue which lowers the IOP, has shown to also improve the retinal blood flow in animals.

**Methods:**

The current study therefore evaluated the retinal vessel density in the peripapillary and macular region of POAG patients with normal IOP treated with topical Tafluprost (*n* = 20) compared to surgically treated patients with normal IOP (*n* = 22) using optical coherence tomography angiography (OCT-A). The retinal flow density was obtained after binarisation and evaluated in five sectors.

**Results:**

There was a significantly higher peripapillary flow density in all sectors in Tafluprost treated eyes when compared to post-surgery eyes. The flow density in the inferior sector of the superficial plexus in the macular region was also significantly higher in the Tafluprost group.

Conclusions: These results indicate that Tafluprost not only lowers IOP, but may also enhance retinal blood flow in POAG patients with a normal IOP.

## Background

Primary open-angle glaucoma (POAG) is a leading cause for blindness worldwide [1, 2]. POAG shows no symptoms in early stages. Instead, patients present a slow, progressive deterioration of nerve fibre layer and increasing visual field losses. Glaucoma often affects both eyes. The pathophysiology of this chronic, multifactorial and neurodegenerative disease remains unclear [1].

An elevated IOP is the most crucial risk factor for POAG as well as disease progression [1]; hence, current treatment options focus on lowering it. However, treating glaucomatous eyes is challenging and requires lifelong and thorough follow-up examinations. Within the therapeutic alternatives, surgical interventions aim to enable improved drainage of the aqueous humour through the implementation of a mechanical drain. Medicamentous treatment involves either reducing aqueous humour production or increasing drainage.

However, the regulation of IOP in POAG does not ensure the stabilization of this irreversible disease and deterioration in the patient’s visual field remains possible. In addition to the mechanical pathophysiological theory of elevated IOP, retinal blood flow is reduced in glaucoma [3]. The damage caused by decreased retinal blood flow might contribute to the development and deterioration of POAG [4, 5]. Vascular dysregulation and the resulting insufficient blood supply may lead to a progressive loss of nerve fibres.

Tafluprost, a selective fluoroprostaglandin (FP) receptor agonist from the group of prostaglandins - used as a topical medication to lower the IOP - appears to offer a potential method for improving retinal perfusion. Animal testing in cats showed increased blood flow in larger retinal vessels following topical application of Tafluprost [6]. In the experimental glaucomatous eyes of monkeys treated with Tafluprost, optic nerve head (ONH) circulation increased significantly after application [7]. Furthermore, Tafluprost improved the ONH blood flow in human preperimetric glaucomatous eyes with ONH impairment [8]. In a case series of 11 patients with treatment-naïve POAG eyes, Tafluprost showed an increased parafoveal retinal blood flow velocity after up to 12 weeks of topical application [9]. In addition to its IOP lowering effects, Tafluprost may offer the possibility of an enhanced retinal perfusion.

Recently, the use of Optical Coherence Tomography Angiography (OCT-A) has been the favoured approach to visualising blood flow changes within the retina. OCT-A offers the possibility of a non-invasive illustration of capillary retinal vessels using the differentiation between moving components and static tissue [10]. Erythrocyte movement allows for acquisition of retinal perfusion in different layers both axially and transversally [11]. In addition to blood flow, retinal structures can also be visualized [11]. Changes within the retinal vascularisation can be found in OCT-A, for example, abnormal vessel formations, missing perfusion or atypical existence of newly formed vessels [10]. Acquisition is performed through undilated pupils. An intravenous implementation of drugs, as used in fluorescence-angiography, is not needed. Though a visualization of leakage is not possible with OCT-A, its easy and user-friendly handling offers many new possible diagnostic options.

Examination of glaucomatous eyes with OCT-A has been a recent topic of interest. Existing studies show a significantly reduced retinal vessel density and blood flow in POAG eyes compared to healthy ones [12–14]. These perfusion differences even allow the discrimination of healthy and POAG eyes through only the vessel density of the ONH acquired with OCT-A [14]. The loss of macula vessel density within 1 year in POAG eyes was significantly faster than in healthy eyes [15]. Vascular changes detected by OCT-A in POAG eyes might either cause optic nerve damage or result from it [16]. An improvement of retinal perfusion might decelerate the progression of POAG. Therefore, medication supporting perfusion could help delay the loss of retinal nerve fibres and visual field. There have been reports indicating that Tafluprost increased blood flow. However, investigating the effect of Tafluprost on retinal blood flow is challenging as intra-patient comparisons of the retinal blood flow before and after the start of IOP-lowering medication could be confounded by the IOP-lowering effect. Therefore, the study intends to evaluate potential differences in retinal vessel density through OCT-A between Tafluprost-treated eyes of POAG patients having reached IOP values between 10 and 21 mmHg either by topical treatment with Tafluprost (*n* = 20) or after successful surgical treatment (*n* = 22).

## Methods

This was a prospective, cross-sectional study performed at the Department of Ophthalmology of the University Medical Centre Hamburg-Eppendorf, after approval of the ethics review board of the medical association Hamburg (registered study number: PV5467) and following the recommendations of the Declaration of Helsinki. Written informed consent was obtained from each patient.

### Study population

Patients with POAG were prospectively included between May 2017 and July 2018. Suitable patients were over the age of 18 years and diagnosed with POAG. Following the proposed definition by the World Glaucoma Association, POAG was defined by a progressive thinned retinal nerve fibre layer thickness (RNFLT) and progressive narrowed retinal rim without mandatory visual field defects [17].

Patients were either topically treated with Tafluprost or had undertaken a pressure-lowering surgery. Applicable surgical treatments were deep sclerectomy, trabeculotomy and trabeculectomy. It is important to note that these patients received surgery due to malcompliance or multiple eye drops intolerance. Therefore, there was only early glaucomatous damage in these selected groups. Patients with retinal pathologies, additional topical medication, inconsistent medication, cardiovascular diseases and poor image quality were excluded. The following data was collected from all patients: age, gender, IOP and standard automated perimetry (SAP). Visual field measurements were evaluated by dividing the illustrated results into three segments (superior, inferior and central) and documenting visual field loss within each segment (yes/no). Mean deviation (MD) was also obtained.

### OCT-A device and scanning protocol

Both study groups received OCT and OCT-A imaging using the Topcon DRI OCT Triton (Topcon Corporation, Tokyo, Japan; Software Version 10.13.003.06). The scans were performed through undilated pupils. For each eye three images were collected: An OCT-A 6 × 6 mm scan field centring the fovea, an OCT-A 6 × 6 mm scan field centring the optic nerve, and an OCT scan (3D Horizontal Wide Scan) of the retinal nerve fibre layer thickness. To assure adequate scan quality and comparability, OCT-A scans required an image quality of ≥60 and had to meet the consensus criteria for retinal OCT quality assessment (OSCAR-IB) [18].

### OCT-A image processing

**Fig. 1:**
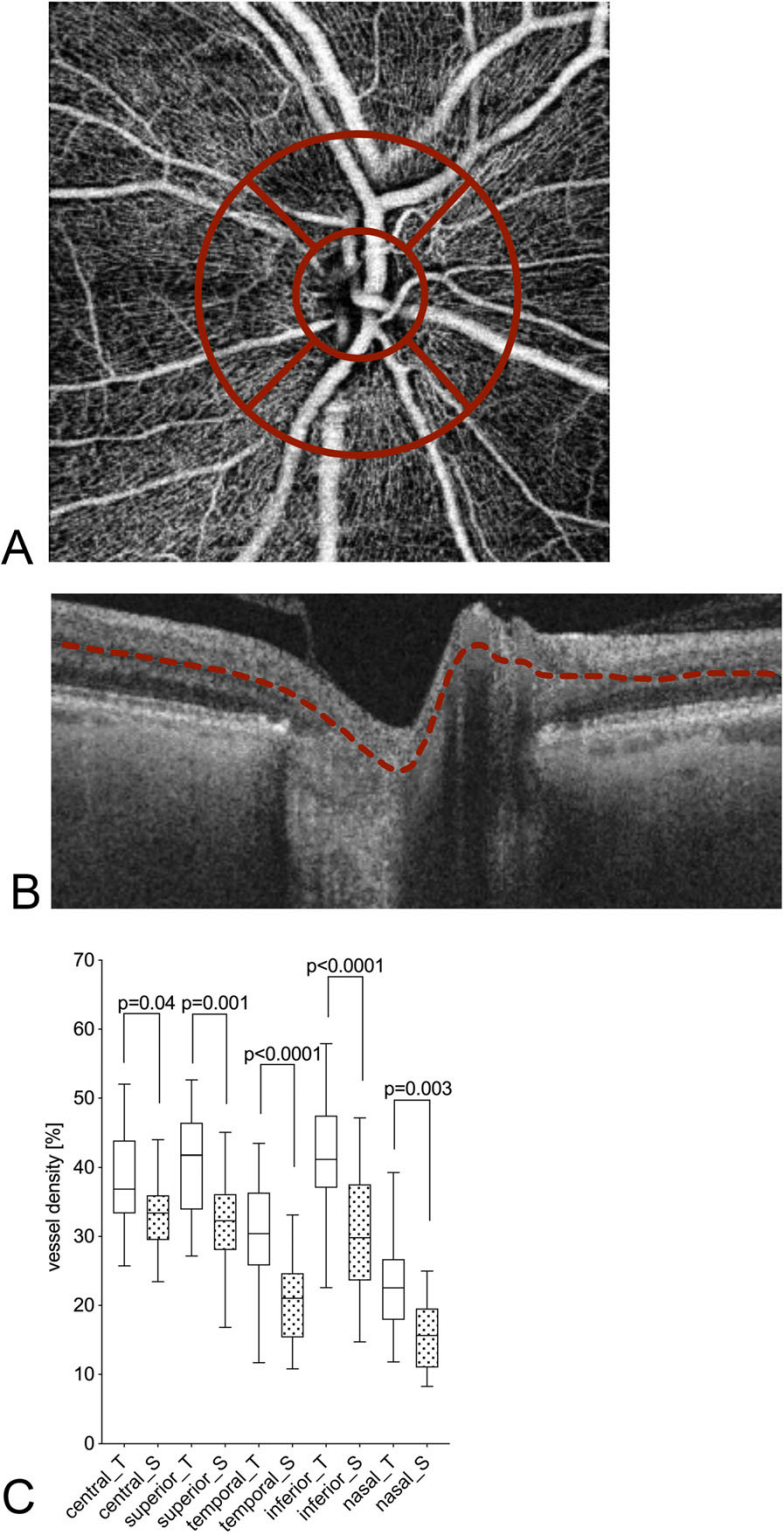
Peripapillary OCT-A flow density [%] for patients with glaucoma undergoing Tafluprost therapy and post-surgery in the optic nerve area. An exemplary en-face OCT-A scan (**a**), OCT scan image (**b**) and the respective vessel density (**c**) are shown. The red dotted line in the OCT scan (**b**) highlights the layers included in the vessel density analysis. The values of patients undergoing Tafluprost therapy are marked with “_T” and white bars; the post-surgery patients with “_S” and dotted bars. The individual sectors are shown (central, superior, temporal, inferior, nasal). Presented *p-*values were calculated using unpaired t-tests

Blood flow analysis was obtained after binarisation. Pixel density was measured via an individual analysis with Matlab®. The scans of the superficial capillary plexus of the peripapillary region and the superficial and deep capillary plexus of the macular region were each divided into five sectors (according to the Early Treatment Diabetic Retinopathy Study (ETDR)): central, nasal, temporal, superior and inferior (central sector: 1 mm diameter; four sectors: 3.5 mm diameter. See Fig. 1a).

### Statistical analysis

The comparison of retinal blood flow between the groups was performed through the parametric t-test with Sidák correction [19]. (Statistical analysis compared the different pixel densities of both groups in the following vessel plexus: the superficial capillary plexus of the macula (from the inner limiting membrane (ILM) + 2.6 μm to the inner plexiform layer/inner nuclear layer (IPL/INL) + 15.6 μm), the deep capillary plexus of the macula (from the IPL/INL + 15.6 μm to the IPL/INL + 70.2 μm) and the superficial capillary plexus of the peripapillary region (from top of the image to the IML + 130 μm). Statistical analysis of age was performed using the Mann-Whitney test. Gender distribution was analysed using the chi-square test with Yates’ correction. SAP MD, defect location and average RNFLT were compared using the independent samples t-test.

## Results

### Study participants

Seventy-one eyes of 42 patients were enrolled in this study. Thirty-three eyes from 20 patients were treated with topical Tafluprost. The post-surgery group included 38 eyes from 22 patients.

### Demographics and ocular characteristics

There were no significant differences concerning age, gender, IOP, SAP and average RNFLT between the groups (Table 1). The mean age was 65.9 ± 11.9 years in the Tafluprost group and 70.3 ± 6.6 years in the surgically treated group. The male/female ratio was 23/10 among the Tafluprost-treated patients and 27/11 in the operated group. The IOP was 14.2 ± 0.5 mmHg in the Tafluprost group and 13.6 ± 0.4 mmHg in the post-surgery patients. SAP MD was − 4.4 ± 6.8 dB in the Tafluprost group and − 5.3 ± 3.8 dB in the post-surgery patients.

**Table 1:** Demographics and ocular characteristics of the study population

Variables	Tafluprost-treated eyes (33 eyes of 20 patients)	Post-surgery eyes (38 eyes of 22 patients)	***p***-value
Age [years]	65.9 ± 11.9	70.3 ± 6.6	0.09 *
Gender (male/female)	23/10	27/11	0.90 †
IOP [mmHg]	14.2 ± 0.5	13.6 ± 0.4	0.33 ‡
SAP MD [dB]	− 4.4 ± 6.8	−5.3 ± 3.8	0.48 ‡
SAP defect location (superior/inferior), n	6/9	15/13	
SAP defect location (central), n	6	9	
Average RNFLT [μm]	82.3 ± 17.2	74.7 ± 20.2	0.11 ‡
HRT cup/disc area ratio	0.40 ± 0.15	0.43 ± 0.18	0.56 ‡

∗ The comparison was performed using the Mann-Whitney test

† The comparison was performed using the chi-square test with Yates’ correction

‡ The comparison was performed using the independent samples t-test

### Flow density

Flow density measured with OCT-A was significantly higher in Tafluprost-treated eyes than in post-surgery eyes.

In the optic nerve area, each sector showed a significantly higher flow density in eyes treated with Tafluprost (central 39.3 ± 11.8% versus 32.8 ± 5.9%, *p* = 0.04; superior 41.1 ± 9.4% versus 32.1 ± 7.4%, *p* = 0.001; temporal 32.1 ± 14.9% versus 20.9 ± 5.9%, *p* <  0.0001; inferior 42.6 ± 12.4% versus 30.4 ± 8.9%, *p* <  0.0001; nasal 23.8 ± 10.1% versus 15.7 ± 4.9%, *p* = 0.03; Table 2 and Fig. 1).

**Table 2:** OCT-A flow density [%] in patients with glaucoma undergoing Tafluprost therapy and post-surgery in different areas/segments: optic nerve area, macular area superficial retinal plexus from the inner limiting membrane to the inner plexiform layer and macular area deep retinal plexus from inner plexiform layer to outer plexiform layer. *P*-values were calculated via unpaired t-tests and adjusted using Sidák correction. Significant *P*-values are printed in bold

Flow density [%]	Tafluprost-treated eyes	Post-surgery eyes	*P*-value
**Optic nerve area**			
central	39.3 ± 11.8	32.8 ± 5.9	**0.04**
superior	41.1 ± 9.4	32.1 ± 7.4	**0.001**
temporal	32.1 ± 14.9	20.9 ± 5.9	**< 0.0001**
inferior	42.6 ± 12.4	30.4 ± 8.9	**< 0.0001**
nasal	23.8 ± 10.1	15.7 ± 4.9	**0.003**
**Macular area, superficial retinal plexus**			
central	11.7 ± 3.7	8.0 ± 2.7	0.78
superior	28.3 ± 13.7	23.3 ± 4.8	0.21
temporal	17.5 ± 8	14.4 ± 3.7	0.89
inferior	26.6 ± 10.5	19.7 ± 3.2	**0.03**
nasal	25.7 ± 8.7	21.3 ± 6.1	0.47
**Macular area, deep retinal plexus**			
central	21.1 ± 6.2	15.5 ± 5.6	0.14
superior	39.5 ± 7.2	34.2 ± 9.3	0.22
temporal	29.8 ± 5.4	25.5 ± 10.4	0.55
inferior	31.1 ± 10.9	27.0 ± 9.2	0.64
nasal	40.4 ± 7.6	35.3 ± 10.9	0.30

**Fig. 2:**
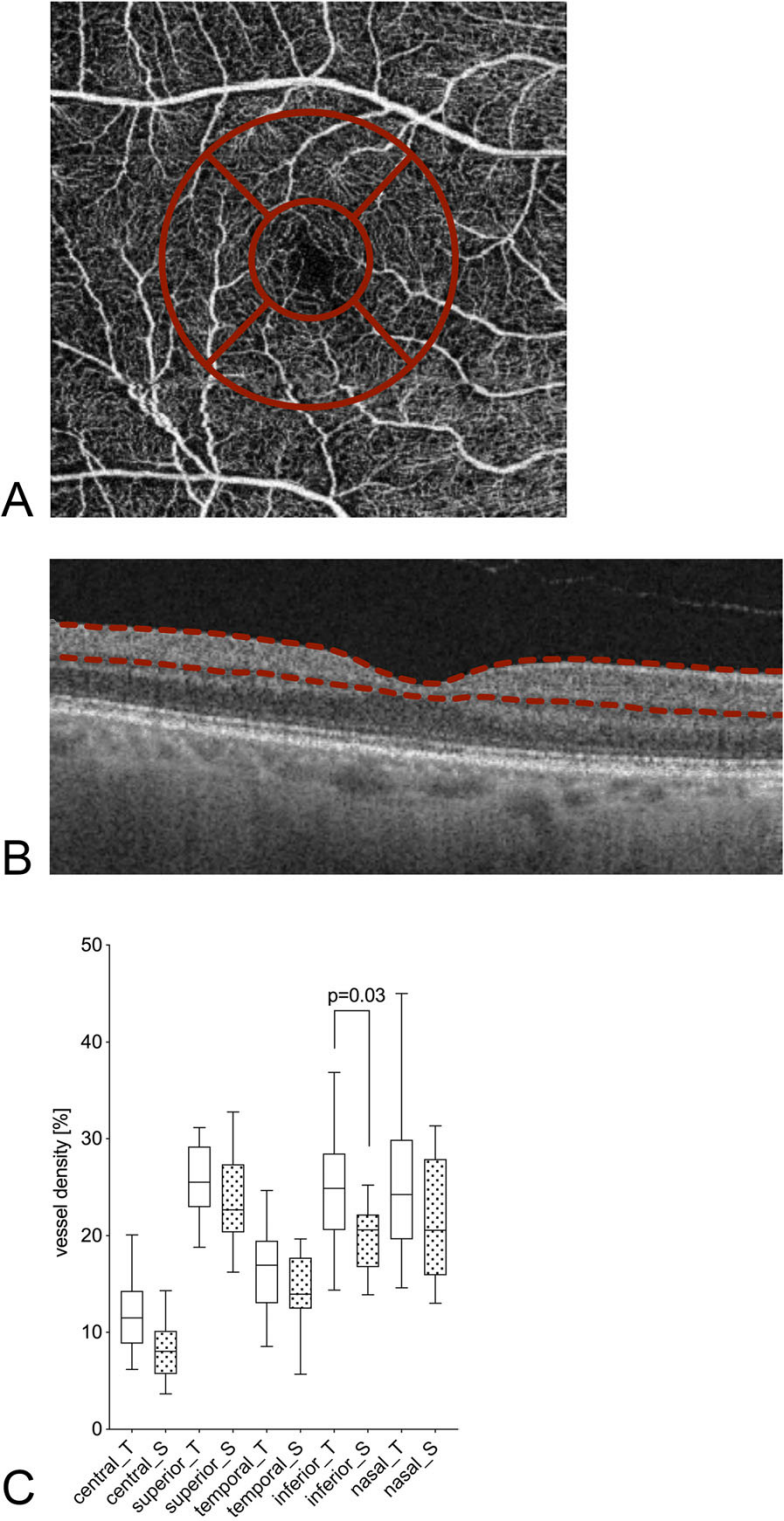
Macular superficial retinal plexus OCT-A flow density [%] for patients with glaucoma undergoing Tafluprost therapy and post-surgery in the optic nerve area. An exemplary en-face OCT-A scan (**a**), with OCT scan image (**b**) and the respective vessel density (**c**) are shown. The red dotted line in the OCT scan (**b**) highlights the layers included in the vessel density analysis. The values of patients undergoing the Tafluprost therapy are marked with “_T” and white bars; the post-surgery patients with “_S” and dotted bars. The individual sectors are shown (central, superior, temporal, inferior, nasal). Presented *p*-values were calculated using unpaired t-tests

**Fig. 3:**
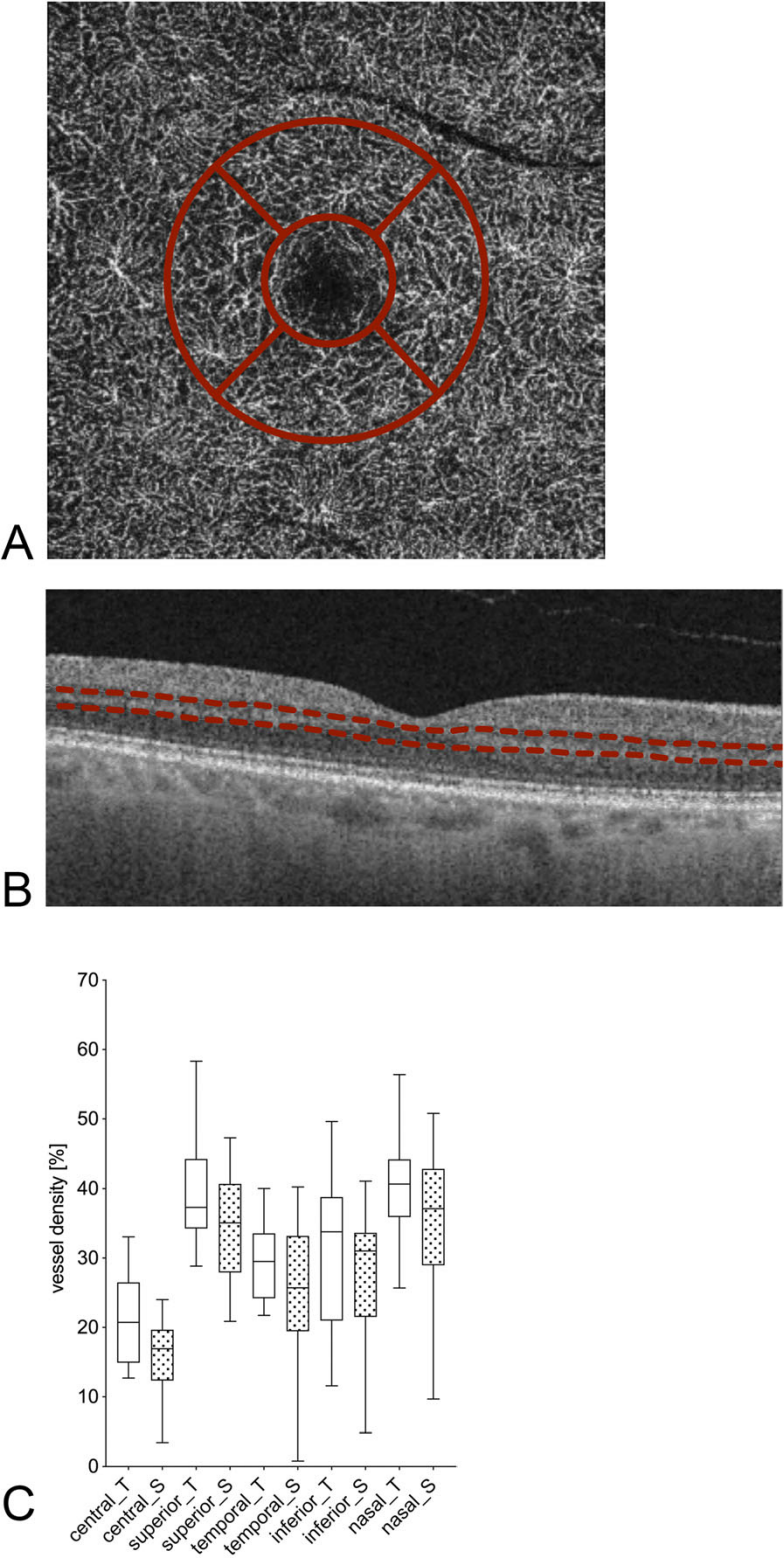
Macular deep retinal plexus OCT-A flow density [%] for patients with glaucoma undergoing Tafluprost therapy and post-surgery in the optic nerve area. An exemplary en-face OCT-A scan (**a**), OCT scan image (**b**) and the respective vessel density (**c**) are shown. The red dotted line in the OCT scan (**b**) highlights the layers included in the vessel density analysis. The values of patients undergoing Tafluprost therapy are marked with “_T” and white bars; the post-surgery patients with “_S” and dotted bars. The individual sectors are shown (central, superior, temporal, inferior, nasal). Presented *p-*values were calculated using unpaired t-tests

The flow density of the macular area was divided into the superficial and the deep retinal plexus: Flow density of the superficial retinal plexus was significantly higher within the inferior sector (26.6 ± 10.5% versus 19.7 ± 3.2%, *p* = 0.03; Table 2 and Fig. 2), while all other sectors showed no significant differences. The deep retinal plexus in the macular area showed no significant differences in flow density between the groups (Table 2 and Fig. 3).

## Discussion

The present study found a significantly higher retinal flow density in all five sectors of the ONH area and in the inferior sector of the superficial retinal plexus in the macular region within Tafluprost-treated POAG eyes compared to non-treated post-surgery POAG eyes (both groups with IOP between 10 and 21 mmHg).

Tafluprost (AFP-168) is a synthesised Prostaglandin F_2α_ analogue binding to the prostanoid FP receptor, which is present in human ocular tissues [20, 21]. This binding to FP receptors stimulates an increased uveoscleral outflow, which leads to an IOP-decrease [21]. Analysis in mice showed a dose-dependent effect with a saturating concentration [21]. With more than 10-fold higher affinity to FP receptors than latanoprost-acid, Tafluprost has also been shown to provide a longer lasting effect [6, 20, 21] and a greater night-time efficacy [22]. Tafluprost lowers IOP, at the latest, 4 hours after application and preserves its effect for at least 24 h [23]. In comparison to other prostanoid FP receptor agonists, Tafluprost demonstrated to induce less local side effects and may provide a more stable lowering of IOP [20, 24]. However, it may also lead to side effects similar to other prostaglandin analogues. These include trichiasis and pigmentation changes of the iris, periocular skin and eyelid [20]. In animal studies, Tafluprost has not only shown a vasodilatation on isolated rabbit arteries [25], but enhanced retinal blood velocity in cats [6], in experimental glaucoma in monkeys [7] and in humans [26].

In Glaucoma patients, ocular blood flow is altered [5, 27, 28]. It is not only mechanical pressure, but both reduced blood circulation and vascular dysregulations leading to reperfusion injury that may affect the incidence and progression of glaucoma [27]. Along with secondary influences, there are primary influences on disease development [5, 27]. For this reason, the retinal perfusion is of great interest in clinical glaucoma studies.

OCT-A offers a new diagnostic approach to image vascular changes in the retina. However, it must be mentioned that comparison between different devices and their vessel density measurements is challenging [29]. Nevertheless, within the collected data of POAG patients, there are consistent results concerning vascular changes when compared to healthy patients. Studies using OCT-A were able to show that glaucoma patients present with an impaired vessel density [12, 14, 30–36] as well as a significantly lower flow index [36, 37] and flow density [38] in the ONH area when compared to healthy subjects. In the macular area, an impaired vessel density [12, 15, 34, 39, 40] and flow density [38, 41] has also been shown in POAG patients when compared to healthy subjects. In mild to moderate glaucoma, Moghimi et al. [42] demonstrated an association between lower macular and lower ONH vessel density, as well as a faster rate of RNFL progression.

Our study aimed to investigate the potential effect of Tafluprost on flow density in POAG patients using OCT-A examination of both the optic nerve head and the macular region in a cross-sectional approach. Hee In et al. demonstrated that lowering the IOP could lead to an increased flow density correlated to the IOP reduction [43]. Therefore, an interventional approach to evaluate the effect of Tafluprost on flow density could be confounded by its IOP-lowering properties. Consequently, we compared the flow density between Tafluprost treated POAG patients and patients with POAG who had received IOP-lowering surgery due to malcompliance or multiple eye drops intolerance.

Our study was able to show a significantly higher flow density in Tafluprost treated eyes, especially within the peripapillary sectors and also in the inferior sector of the superficial retinal plexus of the macular region. Our results concerning the macular region were comparable with the findings of Iida et al. [9], who used adaptive optics scanner laser ophthalmoscopy to demonstrate an enhanced mean parafoveal blood flow velocity in a 12-week period after initiating topical Tafluprost treatment in POAG patients. Nevertheless, there was no significant difference in the mean blur rate in the tissue area of the ONH [9]. This is a discrepancy to our study, where significantly different results were observed - at the ONH in particular. Our findings with regard to the altered flow density of the superficial plexus in the macular region could be linked to visual field defects. A significant association between decreased vessel density and severity of visual field impairment has been shown [33]. Our study did not investigate the association between visual field impairment vessel density as any breakdown into a more specific analysis would not be reasonable considering the relatively small group size.

Our results show a higher flow density and therefore could insinuate an improved blood flow within the retina following from the topical application of Tafluprost. However, two issues must be emphasised: (1) The relevance of the flow density or retinal blood flow in terms of cause or effect for the development of POAG cannot be answered by this study. (2) Furthermore, a direct causal relationship between Tafluprost and flow density cannot be established due to the non-interventional approach of this study and the potential confounding factor of its IOP-lowering properties when used in an interventional study.

The strength of the present study is its status as the first investigating the connection between Tafluprost application in POAG patients and retinal blood flow using OCT-A in a cross-sectional approach. OCT-A is a widely spread, user-friendly, robust, reproducible and, recently, commonly used diagnostic approach in vascular retinal diseases [10, 44]. Both groups studied showed no significant differences concerning demographics and ocular characteristics, making comparison more meaningful. Further, our study followed international procedures and adhered to high quality standards to provide firm results. Patients were examined at least 4 weeks after the start of continual Tafluprost application and a minimum of 6 weeks after surgery to prevent distortion as a result of previously applied medication.

The weakness of the study is that no treatment-naïve flow density measurements were undertaken. It has been demonstrated that changes in IOP can alter flow density [45–47]; hence it would be challenging to differentiate the impact on the flow density induced by pressure differences rather than the chosen treatment. Furthermore, in patients with IOP-lowering surgery, a baseline measurement without treatment was not possible were on topical or systemic treatment before surgery as the refusal of therapy to glaucomatous eyes might be ethically difficult to argue nowadays. Therefore, our study included operated POAG eyes, as they carry similar vascular characteristics as well as a comparable IOP. Further, it has been demonstrated that flow density is significantly correlated with morphological and functional indices, and exhibits diagnostic capabilities comparable to currently employed clinical variables [48]. Consequently, we only included patients who underwent early surgery (e.g. due to multiple eye drops intolerance) to ensure that the disease was similarly advanced in both groups.

## Conclusion

In conclusion, our results indicate that Tafluprost not only lowers IOP, but may also enhance retinal blood flow at a normal IOP. This effect might influence disease progression. A more accurate flow density could be estimated by applying statistical methods such as delta method incriminating all confusing factors (such as age, gender, intraocular pressure, spherical equivalent, physical activity, systemic diseases) [47]. Further studies are needed to investigate the long-term benefits of an improved flow density achieved via topical application of Tafluprost in POAG patients with normal IOP and whether different operations might have a varying impact on the flow density.

## Data Availability

The datasets generated during and/or analysed during the current study are available from the corresponding author on reasonable request.

## Abbreviations

ETDR: Early Treatment Diabetic Retinopathy Study
FP: Fluoroprostaglandin
ILM: Inner limiting membrane
INL: Inner nuclear layer
IOP: Intraocular pressure
IPL: Inner plexiform layer
MD: Mean deviation
OCT-A: Optical coherence tomography angiography
ON: Optic nerve head
OSCAR-IB: O: obvious problems; S: poor signal strength; C: centration of scan; A: algorithm failure; R: retinal pathology other than MS related; I: illumination; B: beam placement
POAG: Primary open-angle glaucoma
RNFLT: Retinal nerve fiber layer thickness
SAP: Standard automated perimetry
